# Is Energy Expenditure or Physical Activity Considered When Energy Intake Is Measured? A Scoping Review 1975–2015

**DOI:** 10.3390/nu13093262

**Published:** 2021-09-18

**Authors:** Marcela González-Gross, Raquel Aparicio-Ugarriza, Sergio Calonge-Pascual, Sonia Gómez-Martínez, Alberto García-Carro, Ana Zaragoza-Martí, Javier Sanz-Valero, Carmina Wanden-Berghe, J. Alfredo Martínez, Ángel Gil, Ascensión Marcos, Luis A. Moreno, on behalf of the Spanish Nutrition Society (SEÑ)

**Affiliations:** 1 ImFINE Research Group, Department of Health and Human Performance, Faculty of Physical Activity and Sport Science-INEF, Universidad Politécnica de Madrid, 28040 Madrid, Spains.calonge@upm.com (S.C.-P.); ag.carro@gmail.com (A.G.-C.); 2 CIBEROBN (Physiopathology of Obesity and Nutrition CB12/03/30038), 28029 Madrid, Spain; jalfmtz@unav.es (J.A.M.); agil@ugr.es (Á.G.); lmoreno@unizar.es (L.A.M.); 3 Immunonutrition Research Group, Department of Metabolism and Nutrition, Institute of Food Science, Technology and Nutrition (ICTAN), Spanish National Research Council (CSIC), 28040 Madrid, Spain; sgomez@ictan.csic.es (S.G.-M.); amarcos@ictan.csic.es (A.M.); 4 Department of Nursing, University of Alicante, 03690 Alicante, Spain; ana.zaragoza@ua.es; 5 Alicante Institute for Health and Biomedical Research (ISABIAL-FISABIO Foundation), 03010 Alicante, Spain; carminaw@telefonica.net; 6 Área de Divulgación e Investigación y Servicios, Escuela Nacional de Medicina del Trabajo, Instituto de Salud Carlos III, 28029 Madrid, Spain; fj.sanz@isciii.es; 7 Department of Nutrition, Food Science, Physiology Navarra University, 31009 Navarra, Spain; 8 IMDEA Food, 28049 Madrid, Spain; 9 Department of Biochemistry and Molecular Biology II, and Institute of Nutrition and Food Sciences, University of Granada, 18001 Granada, Spain; 10 GENUD Research Group and Instituto Agroalimentario de Aragón (IA2), Universidad de Zaragoza, 50001 Zaragoza, Spain; 11 Instituto de Investigación Sanitaria Aragón (IIS Aragón), 50009 Zaragoza, Spain

**Keywords:** dietary records, dietary surveys, food diaries, energy expenditure, physical activity, assessment

## Abstract

The health-transitions humans have delivered during the 20th Century associated with the nutrition is that from undernutrition to obesity, which perseveres in the current years of the 21st Century. Energy intake (EI) is a contributing factor and therefore a fascination in nutritional sciences. However, energy expenditure (EE) has not been usually considered as a conjoint factor. Thus, this study aimed to review if studies on adults consider data on dietary intake, specifically EI, and included data on EE and physical activity (PA). A search of MEDLINE from 1975 to December 2015 was managed. Our scoping review consisted of keywords related to EI, dietary allowances, and nutritional requirements. From 2229 acknowledged articles, 698 articles were finally taken fulfilling inclusion and quality criteria. A total of 2,081,824 adults (53.7% females) were involved, and most studies had been conducted in EEUU (241), Canada (42), Australia (30), Japan (32), and Brazil (14). In Europe, apart from UK (64), the Netherlands (31) and France (26) led the classification, followed by Sweden (18), Denmark (17), and France (26). Mediterranean countries are represented with 27 studies. A total of 76.4% did not include EE and 93.1% did not include PA. Only 23.6% of the studies contained both EI and EE. A large methodological diversity was perceived, with more than 14 different methods regarding EI, and more than 10 for EE. PA was only analyzed in scarce articles, and scarcely considered for interpretation of data and conclusions. Moreover, PA was often measured by subjective questionnaires. Dietary surveys show a large diversity regarding methodology, which makes comparability of studies difficult. EE and PA are missing in around 80% of studies or are not included in the interpretation of results. Conclusions regarding EI or diet adequacy in adults should not be taken without analyzing EE and PA.

## 1. Introduction

One of the health-transitions humans have passed during the 20th Century related to nutrition is, with no hesitation, from malnourished to obesity [1], which is continuous in the ongoing years of the 21st Century [2]. During the 1980s of the last century, this transition started and increased steadily, first in some countries such as the EEUU and later in Europe and in developing countries [3]. Obesity has been explained as a multifactorial disease [1] and it is also a complex pathology which includes behavioral, environmental, and genetics interactions [4]. Research in the last years has introduced new contributing factors, such as genetics, physical inactivity, epigenetics, microbiota, social issues [5]. Interestingly, at least 107 factors have been identified as contributors to energy balance [6], which in turn affect body composition and body weight homeostasis. Both energy intake (EI) and energy expenditure (EE) are included, sometimes controversially, as there is no consensus among scientists if the main contributing factor is an increase in EI, a decrease in EE, or a combination or interaction of both. Frequently, EE assessment helps to estimate nutrient requirement [7].

EE is also linked to physical activity (PA), the most variable part of EE [8] as PA increases EE above resting levels [9]. PA has an interactive role with nutrition, and PA, additionally, influences energy balance beyond EE. PA can modify appetite which can affect total EI [10]. During the recent years, the scientific literature has analyzed data about EI and how people eat; however, EE and PA were often not considered, despite both parameters being essential in the global context of raising prevalence of non-communicable diseases, such as obesity, diabetes, and cardiovascular disease [11]. In this sense, the World Health Organization highlighted the necessity to promote PA as a public health issue [12] as it directly related to less cardiometabolic diseases, diabetes, or obesity [13].

Even if data are not available for many countries, longitudinal data have shown a steady decrease in all domains of PA (labor, transport, leisure, homework) for USA, UK, China, Brazil and India [14] and an insufficient PA levels are worldwide increasing [15]. Thus, the evaluation of EE and PA together with EI is necessary when some of these dimensions are evaluated to obtain more accurate data.

On the other hand, advances in nutritional sciences have been growing, and studies have been increasingly published. Standardization in methodologies for field work has been proposed [16,17], but a global consensus and a standard methodology when publishing results is lacking. Concerning EE and PA, it is important to note that these parameters are different constructs and that there are a huge variety of methods (objective and subjective) to evaluate them [11].

Therefore, a scoping review was performed with the aim of analyzing if studies including data on EI also included data on EE and PA. A secondary aim has been to analyze the methodology used in the assessment and those countries where the studies have been performed.

## 2. Materials and Methods

### 2.1. Search Strategy and Data Sources

A scoping review search of MEDLINE from 1975 to December 2015 was performed. Our literature contained keywords related to energy intake and nutrition (“nutritional requirements”, “recommended dietary allowances”, or “diet records”). The main Medical Subject Headings (MeSH) were used as Major Topic to obtain results (references) with a high relevance. The used MeSH and MaJR terms were built up using a systematized search and the final equation was: (“Recommended Dietary Allowances” [Majr] OR “Nutritional Requirements” [Majr] OR “Energy Intake” [Majr] OR “Diet Records” [Majr]) NOT (“Diseases Category” [Mesh] OR “Pregnancy” [Mesh] OR “Breast Feeding” [Mesh]). 

On MEDLINE, an initial screening of MeSH terms was performed to detect relevant keywords. To ensure more complete results in the adults’ population, “humans” and “19+ years” were added as “filters”. This resulted in 2229 results, which included relevant articles and the first article which accomplished our criteria was in 1975. Narrowing down the search with the use of subheadings, such as “diseases category”, “pregnancy”, and “breast feeding” for the MeSH term about nutrition requirements, was considered to remove articles about the effects of pathologies, pregnancy, and breast feeding have on energy intake.

### 2.2. Exclusion Criteria

Articles were excluded if they were written in a language different from English, Spanish or German; if performed on non-healthy people; if subjects were <19 years and when results were not split by age; if they were not original (reviews, comments to the editor, communications); if study subjects were vegetarians; animal studies; if they did not include results about total energy intake; if not published as full-text reports, or studies where the original article was not found.

### 2.3. Search Summary

**Figure 1:**
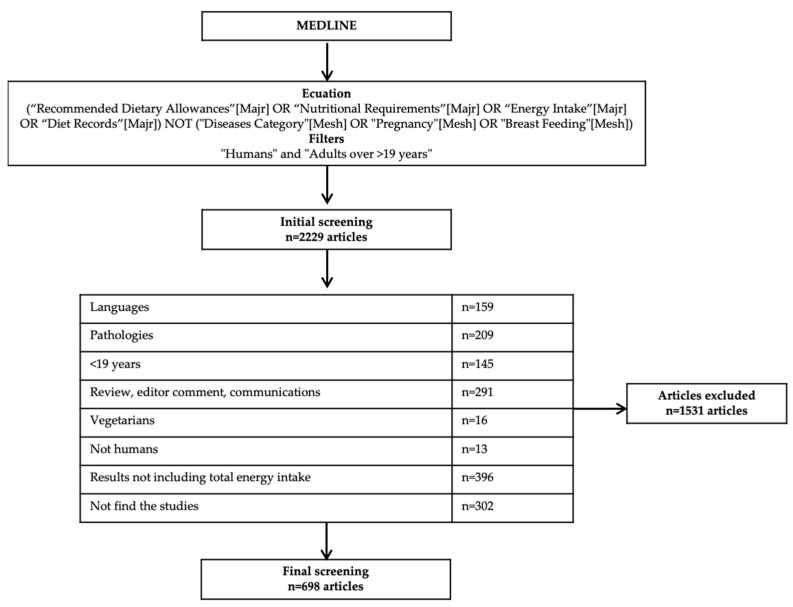
Flowchart of the study selection.

Five researchers (R.A.-U., A.Z.-M., S.C.-P., A.G.-C., and S.G.-M.) individually assessed titles and abstracts by pairs. Inter-reviewer disagreements were elucidated by consensus together with an expert (M.G.-G). The database search retrieved 2229 records in Medline. In total, 172 were written in other languages different from English, Spanish and German. After screening by the abstracts, 209 articles were found that included pathologies and were excluded. A group of 145 articles were excluded because the filter “>19+ years” was not met. Moreover, 291 articles were reviews, comments to the editor or communications. Sixteen articles and 13 articles were performed on vegetarian population and animals, respectively, and therefore, were excluded. After reading the full article, 396 studies were excluded because results about energy intake were missing. Additionally, it was not possible to get the full article of 302 studies. After assessing full-text articles for eligibility and performing the quality assessment, 698 articles were finally held to be included in the scoping review (Figure 1). Each of the articles were analyzed independently by two researchers. Final decisions were taken altogether.

To facilitate the handling of such a high number of articles, we classified them into 8 categories (Body composition, Drugs, Energy Balance, Nutrients, Appetite, Physiology, Food pattern, Methodological). These convenience categories were chosen only for organizational/logistic reasons considering the main aim of the study and should not be considered as completely comprehensive. In fact, many articles could have been included in more than one of these categories. The categorization design does not affect the results of our review.

## 3. Results

Table 1 summarizes the main results of all reviewed studies by categories. 698 studies that met our inclusion criteria. From these studies, only 23.6% included EE and 6.9% included PA measurements. Interestingly, from the total of 2,082.824 adults, more females (53.7%) were analyzed than males. In several studies, sex of the subjects was not specified; therefore, the sum of males and females does not sum up the total number. Complete tables including all analyzed studies can be found in Supplementary Materials. 

Regarding EI assessment, the most used methods were food frequency questionnaire (FFQ), 24 h dietary recall (24 h DR), food diary, followed by food record (FR) and weighed food (WF).

The main methods employed to measure EE were indirect calorimetry (IC) and double labelled water (DLW), followed by diverse and mainly not specified questionnaires. Other methods were accelerometers and the use of different equations proposed by authors such as Ainsworth et al. (1993) [18], Schofield et al. (1985) [19] and Harris and Benedict (1919) [20]. Furthermore, most studies measured EE of specific activities and they did not present results on total EE.

PA was mostly evaluated by means of non-validated questionnaires, validated questionnaires like IPAQ (short and long version) or Minnesota questionnaire and questionnaires used in specify studies as EPIC and NHANES.

We would like to highlight some additional aspects that we have observed. In 321 studies (46%), the date of data collection was not mentioned, which makes it impossible to know the time passed between data collection and publication. Additionally, 137 studies (19.6%) did not mention the food composition table used for calculating energy (and nutrient) intake. Different names for the same method in the literature (dietary record, food record, food diary) were identified. Most of the studies did not consider PA of subjects to enroll them in the study nor did they give instructions regarding their PA during the study if it was an intervention. Additionally, most of reviewed studies did not consider this variable in the interpretation of data and conclusions.

**Figure 2:**
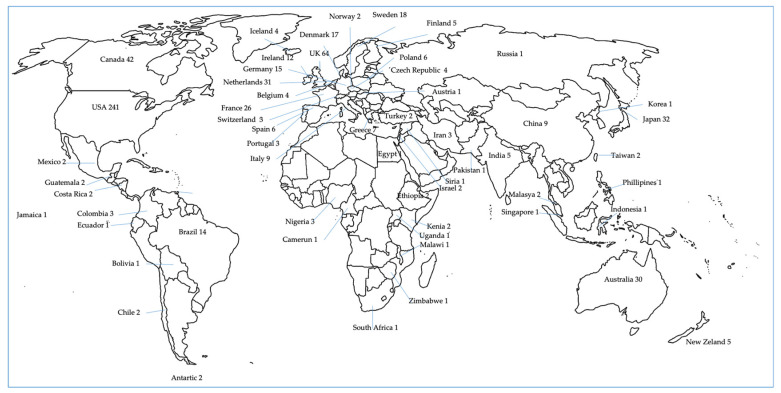
Country origin of the reviewed articles and number.

Figure 2 shows the countries in which the studies included in this review were performed. By countries, highest number of studies took place in EEUU, followed by United Kingdom, Canada, and Australia. In Europe, Northern European countries (without UK, 118 studies) are more represented than Southern European countries (27 studies). In Asia, from a total of 61 studies, most are performed in Japan. Twenty-five studies were completed in South America, with Brazil being the leading country. Africa is the continent least present in this review in comparison with other continents. For Spain, no articles were included analyzing both EI and EE. Only a few studies were conducted in Africa in comparison with other continents.

## 4. Discussion

This is the first time that a scoping review is performed aiming at analyzing if nutritional studies which include data on EI also include data regarding EE and PA. Exclusion criteria have been kept low to include the highest number of eligible articles, even if this has contributed to a huge amount of work when interpreting the studies. The main result is that only 23.6% of studies included data on EE and 6.9% data on PA. This means that in most of nutritional studies, only one side of the energy balance equation has been involved and analyzed.

PA and EE have been measured in few studies and mostly by non-validated questionnaires. It is a fact that standardization started in the 1990s with Ainsworth publication regarding METs [18], proposing a coding scheme for classifying PA by rate of EE. The international group that came together to develop the IPAQ gave an important push forward towards comparability of results [21]. WHO launched the GPAQ in 2006 with the aim to produce valid and reliable estimates of PA [22], especially relevant to developing countries arguing that patterns of EE differ from developed countries. However, even in the 1980s, authors such as Baecke et al. (1982) [23], Bouchard et al. (1983) [24], and Folsom et al. (1986) [25] already proposed questionnaires in order to standardize PA data collection and EE calculation. So it is not understandable that a high number of studies develop their own questionnaire of PA, and even less, that PA is often assessed by a single question [26]. Objective methods such as pedometers and accelerometers have been introduced more recently [27] and are still scarce in the reviewed studies. Nutrition researchers must be aware of the methodology available to measure PA and they need to demand the same standards in quality and accurateness as on the dietary intake part. A varied approach that mixes both the objective and subjective methods involving novel devices and electronic capture of PA questionnaires has been proposed as valid for most nutritional surveys [28].

PA is a known factor to influence appetite [29,30]. Quite a consensus exists in regard to acute exercise reducing appetite, as summarized in the meta-analysis of Schubert et al. (2014) [31], decreasing appetite stimulating hormones such as ghrelin and increasing appetite inhibiting hormones such as PYY, in turn reducing significantly relative post-exercise EI [32]. Less data is available on the long-term effect of regular PA on appetite and EI, authors stating that habitual PA improves appetite control and that physically active subjects compensate energy deficit differently than inactive subjects [33,34]. If this deficit is because of exercise or due to reduced dietary intake, it adds even more complexity to interpret this topic. In their systematic review, Beaulieu et al. (2016) propose that there is a J-shaped relationship between PA level and energy intake [35]. On the other side, physical inactivity has been proposed to be a source of overconsumption and appetite dysregulation [36]. PA should be considered in this kind of studies as it can act as confounder and lead to wrong conclusions. In a recent study performed in adults, authors conclude that EE, specifically EE due to PA, *per se* exerts influence over daily food intake, with both metabolic (RMR) and behavioral (AEE) components of total daily EE potentially influencing EI via their contribution to daily energy requirements [37]. In this sense, some years ago our group analyzed this relationship in data from the HELENA study. Physically active adolescents presented a higher EI intake and lower body fat than their non-active counterparts [38].

Nutrient adequacy and food patterns have been linked to PA, with a higher percent of subjects meeting DRIs with increased PA level [39]. In a study performed in elderly Spanish subjects, higher amounts of water and other beverages were consumed by those being active and low sedentary than in the other PA groups [40]. Positive metabolic effects of regular PA are explicitly demonstrated in the scientific literature [41]. In an experimental study in which adults were overfed (additional 1500–2000 kcal/day), PA was able to inhibit the expected deterioration in glucose and fat metabolism [42]. 

PA is also a known factor to influence body composition. In fact, a physically active lifestyle has been associated with lower body weight and fat [8,43] and an increase in muscle mass and density [44]. On the contrary, a decrease in PA and sedentary behaviors is positively associated with adiposity [36,45]. Drenowatz et al. (2015) performed a prospective study suggesting a reciprocal association between the time spent in MVPA and body fat content [46]. Interestingly, body fat distribution seems to also be determined by the interplay between PA and EI [42], and regular PA can prevent increases of visceral adipose tissue even in the presence of overeating. These authors highlight that there is a complex interaction between body composition, diet, and PA and that more studies are needed. However, it is also urgent to establish a standardized methodology in nutritional studies in which PA should be considered among inclusion/exclusion criteria and as a confounder in intervention studies. Additionally, daily PA and EE should be included in the descriptive of the subjects, and in the interpretation of data. These were also part of the conclusions of the Expert Meeting about “Methodology of dietary surveys, studies on nutrition, PA and other lifestyles” held in Laguardia (Spain) in 2014 [16]. Additionally, harmonized methods should be used to assess PA and EE in the sense already reported in a former review [47].

We have performed a 40-year review of nutritional studies and there is no clear time-trend observed. Some early studies included EI and EE [48,49], whereas studies published more recently did not. Popkin and Nielsen (2003) conducted a study from 1962 to 2000 and the main objective was to explore trends in caloric sweetener intake, the role of urbanization and income changes in explaining these trends, and the contribution of specific foods to these changes in the United States [50]. Nevertheless, they did not examine the effect of PA. O’Brien et al. (2015) analyzed the evolution of food patterns and portion size in Irish adults including several influencing factors (sex, age, body mass index and social class), but PA nor EE were not included [51]. Interestingly, Imada et al. (2014) specifically state the influence of PA on appetite, but do not include PA analysis in their study [52]. Park et al. (2014) analyzed the association between diet quality and irisin levels and did not include any PA nor EE measurement in their study [53]. Interestingly, Kishi et al. (1983) indicated in their study in 1983 that lack of consideration of energy intake in N balance studies may lead to erroneous conclusions [54].

Some additional aspects which were not a primary aim of our review but that came up during the work are worthwhile to be mentioned. At least one-quarter of the studies did not include the date of the field work, so time elapsed between data acquisition and publishing cannot be established. Data from Africa, Asia, South America, and Mediterranean countries are underrepresented in the studies included in this review. Even if we cannot extrapolate to the whole body of publication in nutritional sciences, at least the doubt arises if data from Anglo-Saxon countries should be the baseline for global nutritional measures. A similar observation related to scientific production about hospital-based home care services was made by Sanz-Vareo and Wanden-Berghe (2017) [55], as most documents included in the review were written in English and came mostly from US institutions. Regional and country-specific studies and reviews are needed to establish local nutritional policies in most parts of the world.

**Strengths and Limitations**

As our scoping review has followed the methodology proposed to be systematic, this is one of its strengths. The high number of articles included in the review makes it possible to analyze the evolution of nutritional sciences and relationships with PA. The lack of consensus regarding methods used for assessment and even regarding the names of the methods in the reviewed articles can have led to some misclassification but should not influence the main outcome of our review.

## 5. Conclusions

A huge diversity related to the methodology was experimental, which makes comparability of studies challenging. EE and PA are misplaced in around 80% of articles or are not incorporated in interpretation of outcomes. EI should not be taken deprived of analyzing EE and PA. PA and EE should be included in the subject’s description in all studies. We emphasize the need for a methodological agreement and standardization concerning dietary intake and EE in nutritional studies.

## Figures and Tables

**Table 1:** Descriptive characteristics by categories.

Category	Body Composi-tion	Drugs	Energy Balance	Nutrients	Appetite	Physio-logy	Food Pattern	Methodo-logy	Total
n (male/female)	16.864 (3.013/3.342)	6.838 (2.589/ 2.700)	352.354 (26.462/ 326.417)	48.099 (19.427/ 328.853)	34.467 (9.082/ 24.241)	2440.573 (2.443/ 188.059)	929.342 (14.745/ 152.140)	340.393 (142.328 180.767)	2081.824 (449.606/ 1117.708)
Number of studies (n, %)	8 (1.1%)	10 (1.4%)	100 (14.3%)	55 (7.9%)	98 (14.0%)	98 (14.0%)	179 (25.7%)	150 (21.6%)	698 (100%)
Articles which included EE (n, %)	2 (1.2%)	2 (1.2%)	76 (46.1%)	5 (3.0%)	15 (9.1%)	18 (10.9%)	12 (7.3%)	35 (21.2%)	165 (23.6%)
Articles which included PA (n, %)	1 (2.1%)	0	10 (20.8%)	1 (2.1%)	2 (4.2%)	29 (60.4%)	5 (10.4%)	0	48 (6.9%)

EE: Energy expenditure; PA, physical activity. Note: the total number of participants and the sum of each category is different as not all the article includes the number of participants in each sex.

## Data Availability

Not applicable.

## Supplementary Materials

The following are available online at https://www.mdpi.com/article/10.3390/nu13093262/s1, Excel data review results.
